# Sparse Asymmetry in Locus Coeruleus Pathology in Alzheimer’s Disease

**DOI:** 10.3233/JAD-231328

**Published:** 2024-04-30

**Authors:** Elise Beckers, Joost M. Riphagen, Maxime Van Egroo, David A. Bennett, Heidi I.L. Jacobs

**Affiliations:** a Faculty of Health, Medicine and Life Sciences, School for Mental Health and Neuroscience, Alzheimer Centre Limburg, Maastricht University, Maastricht, The Netherlands; b GIGA-Cyclotron Research Centre-In Vivo Imaging, University of Liège, Liège, Belgium; c Athinoula A. Martinos Center for Biomedical Imaging, Department of Radiology, Massachusetts General Hospital, Boston, MA, USA; d Harvard Medical School, Boston, MA, USA; e Rush Alzheimer’s Disease Center and Department of Neurological Sciences, Rush University Medical Center, Chicago, IL, USA

**Keywords:** Alzheimer’s disease, asymmetry, autopsy, brainstem, locus coeruleus, neurons, tangles

## Abstract

Tau accumulation in and neurodegeneration of locus coeruleus (LC) neurons is observed in Alzheimer’s disease (AD). We investigated whether tangle and neuronal density in the rostral and caudal LC is characterized by an asymmetric pattern in 77 autopsy cases of the Rush Memory and Aging Project. We found left-right equivalence for tangle density across individuals with and without AD pathology. However, neuronal density, particularly in the caudal-rostral axis of the LC, is asymmetric among individuals with AD pathology. Asymmetry in LC neuronal density may signal advanced disease progression and should be considered in AD neuroimaging studies of LC neurodegeneration.

## INTRODUCTION

**Fig. 1:**
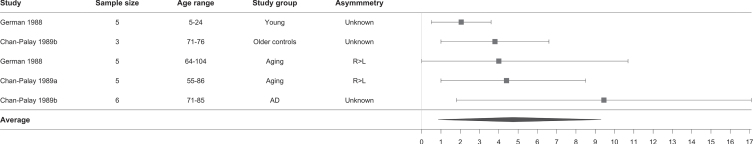
**Overview of asymmetry in locus coeruleus neuronal count in autopsy studies.** Visualization of the reported left-right differences in neuronal count in the LC in autopsy studies [7–9]. The square indicates the mean percentage left-right difference in neuronal count with the bars representing the minimum and maximum reported percentage asymmetry in neuronal count of the LC. The diamond provides the average of all studies (4.74%, range: 0.86% – 9.30%). Unknown asymmetry means that percentage difference in asymmetry was reported without providing the directionality. The reference of Chan-Palay 1989a refers to reference [8], while Chan-Palay 1989b refers to reference [7].

Findings from autopsy and imaging studies established that the locus coeruleus (LC) accumulates hyperphosphorylated tau and undergoes morphological changes early in Alzheimer’s disease (AD) progression, supporting a critical role for the LC in early detection of AD [1–4]. Even though, the LC modulates many cognitive functions and behaviors, including those affected in AD [5], the evidence regarding potential asymmetry in LC pathology remains ambiguous as autopsy studies often examine only one side of the brain. Immunohistochemistry studies report no morphological asymmetry in LC shape or length in clinically normal cases [6], but reported length differences up to 15.2% in AD cases [7]. Similarly, the count of LC neurons is overall symmetric [8, 9], but neuronal loss becomes more asymmetric in AD (left-right differences in neuronal count up to 8% in clinically normal and 17% in AD; Fig. 1) [7]. Neuronal degeneration occurs as the disease progresses, but importantly, is preceded by accumulation of hyperphosphorylated tau. Beyond the anecdotal report of Braak and Del Tredici (2015) that an asymmetrical pattern of abnormal tau inclusions is seldom observed [10], no quantitative data on (a)symmetry of tau in the LC in AD is available. Recently developed MRI-based measures of LC integrity presumably reflect neuronal density and tangle-related processes [3, 11]. In asymptomatic individuals the left LC exhibited higher integrity values than the right [12–14], but inconsistencies exist in the AD neuroimaging literature, ranging from higher right dorsal LC integrity [12], to no left-right differences [15]. Thus, understanding patterns of pathology in the LC will facilitate the interpretation of MRI-based LC findings during AD progression [3], and contribute to understanding disease heterogeneity, contralateral functional compensation [16], and neuroanatomical correlates underlying resilience [17] or correlations with specific behavioral outcomes. Leveraging the neuropathologic data from the Rush Memory and Aging Project (MAP), we set out to determine whether asymmetry in LC tangle or neuronal density was present in older individuals with and without evidence of AD pathology.

## METHODS

### Participants

The dataset included 77 older participants from the Rush Memory and Aging Project (MAP), a clinical-pathologic observational cohort that started in 1997 [18]. Eligibility criteria included older age, absence of a previous dementia diagnosis and consent to annual clinical evaluation and brain autopsy at death. This sample included individuals for whom detailed LC neuropathology data was available and consisted of individuals with no cognitive impairment (*n* = 29), mild cognitive impairment (*n* = 27) or AD dementia (*n* = 21). At time of death, select clinical data (cognitive history, neuropsychological evaluation and clinical judgment) was reviewed by a neurologist, blinded to postmortem data, who provided a clinical diagnosis based on the National Institute of Neurological and Communicative Disorders and Stroke and the AD and Related Disorders Association (NINCDS/ADRDA) criteria [19–21]. The average time between the last visit and death was 0.77 years (SD = 0.60). All data were de-identified and shared with a Data User Agreement. The study was approved by an Institutional Review Board of Rush University Medical Center. All participants signed an informed consent, an Anatomical Gift Act, and a repository consent which allowed their data to be shared.

### Neuropathological measures

Neuronal density (per mm^2^) and paired helical filaments (PHF) tau tangle density of the LC were examined using immunohistochemistry with a monoclonal anti-tyrosine hydroxylase antibody and an anti-PHF tau antibody AT8, respectively, each at the left or right side of the pons and two levels of the LC, rostral and caudal [22–24]. In addition, tangle density was divided by neuronal density and expressed as percentage. We selected participants who had neuropathologic data on both sides of the LC and both sections (*n* = 77). Using available information on cortical neurofibrillary tangles (Braak) and neuritic plaques (CERAD), the likelihood of AD pathology was identified according to the modified National Institute of Aging (NIA)-Reagan diagnosis of AD and grouped into not present (no or low likelihood) and present (intermediate or high likelihood). This evaluation was performed independent of clinical information [21].

### Statistical analyses

Statistical analyses were performed in R (version 4.1.2, http://www.r-project.org/). Group characteristics are represented in mean and standard deviation or proportion. Asymmetry in tangle density, neuronal density or relative tangle density were related to age, postmortem interval and sex with Repeated Measures ANOVA interacting the relevant variable with the within-factor (hemisphere), including Greenhouse-Geiser correction. Asymmetry in LC pathology measures was tested with paired *t*-tests per LC section (False-Discovery Rate adjustment at α= 0.05 per section) and if non-significant, followed up with the bootstrapped two one-sided test (TOST) procedure for pairwise comparisons (5,000 bootstrap replicates) at α= 0.05. Given that absence of asymmetry evidence (non-significance) does not equate evidence of symmetry, the TOST evaluates whether left and right differences in LC pathology can be considered statistically equivalent to zero or below the smallest effect meaningful for asymmetry (Supplementary Figure 1) [25]. Because of the lack of literature on left-right meaningful differences in LC pathology, we tested left-right differences iteratively across a range of values to detect the highest bound at which equivalence was no longer met: 1 to 10 tangles, 1 to 77 neurons per mm^2^, and 1 to 30% relative difference. Upper limit of the asymmetry equivalence bound was determined by the maximum observed difference for that measure. Tests for asymmetry were performed for the entire LC and for rostral and caudal sections. We then assessed whether left-right asymmetry was equivalent between rostral and caudal LC sections using Repeated Measures ANOVA with two levels (hemisphere and section) with Greenhouse-Geiser and Tukey-adjustment. If non-significant, these analyses were followed up by the TOST. Sensitivity analyses tested asymmetry differences within individuals with and without evidence of AD pathology (NIA-Reagan diagnosis of AD).

## RESULTS

**Fig. 2:**
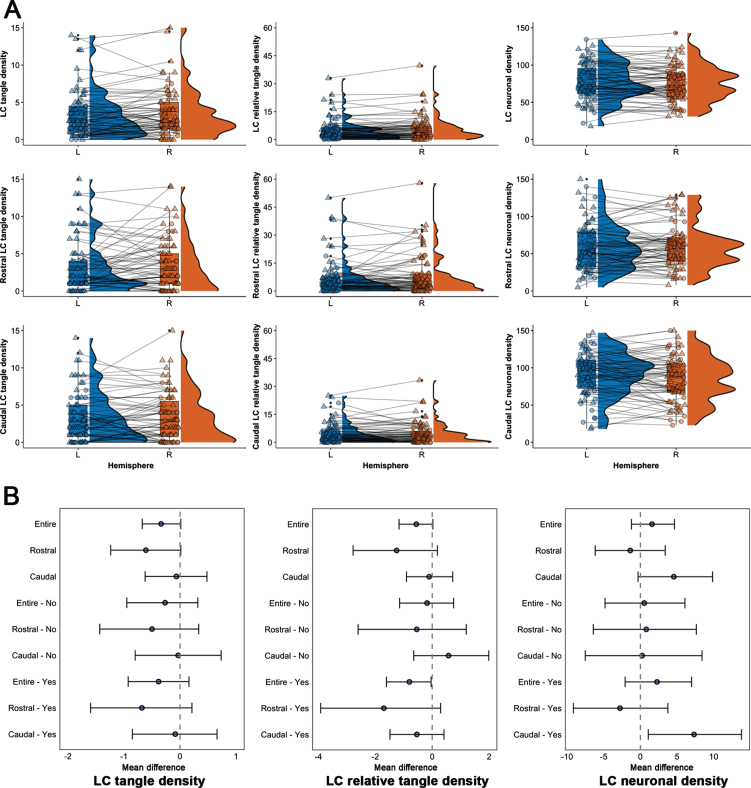
Distribution and effect sizes of left-right differences in the LC measures. A) Boxplots (with median and interquartile range indicated with the horizontal line and bars) and distributions (half violin) depicting the left and right distribution of the LC pathology measures (tangle density, relative tangle density, neuronal density) across the entire LC and its rostral and caudal sections. Triangle shapes indicate individuals with underlying AD pathology according to the NIA-Reagan criteria, whereas circles are those individuals without evidence. B) Mean difference in asymmetry (left-right) for every pathology measure, LC section and group (Yes: evidence of underlying AD pathology; No: no evidence of underlying AD pathology). Error bars represent the 95% confidence intervals (Bootstrapped at 5,000 replicates; α= 0.05).

The average age at death was 88.6 years (range 74.8–99.7), and participants were highly educated, with the majority being female (72%) and 13% carrying one or more *APOE* ɛ4 alleles (Table 1). Left-right differences in LC pathology measures were not associated with age, postmortem interval, or sex (Supplementary Table 1). There was no difference between left and right LC in terms of tangle density, neuronal density, or relative tangle density for the entire LC, the rostral or caudal sections (Supplementary Table 2 for statistical details). Sensitivity analyses revealed no differences in LC measures for individuals with or without evidence of underlying AD pathology, except for an at trend-level asymmetry in caudal LC neuronal density in individuals with AD pathology (on average 7.32 fewer neurons per mm^2^ in the right caudal LC (mean difference: 7.32, t(46) = 2.24, *p* = 0.03, p_FDR_ = 0.09); Fig. 2, Supplementary Table 2). Based on the literature (Fig. 1), > 9.30% difference between left and right would represent an above average asymmetry in older individuals and AD patients, which translates to an asymmetry difference of about 7 neurons per mm^2^; 74.47% of the individuals with AD pathology exhibited a left-right difference of at least 7.32 neurons per mm^2^. TOST-evaluation indicated that rostral tangle density asymmetry was no longer be equivalent at [– 1,1] tangle density. For the neuronal density, left and right was not equivalent at [– 5,5] for the entire LC, [– 6,6] for the rostral LC and [– 9,9] for the caudal LC. For relative tangle density, the null hypothesis of equivalence was rejected at [– 1%, 1% ] for the entire LC and [– 2%, 2% ] for the rostral LC (Supplementary Figure 2). Equivalence bounds for the entire LC and its sections were similar for both the group with and without evidence of underlying AD pathology. These results provide evidence for equivalence (no asymmetry) in LC pathology, except for the caudal neuronal density where the effect exceeded the smallest meaningful difference of 7 neurons per mm^2^.

**Table 1:** Demographics

	MAP (*N* = 77)
**Age at death (y)**	88.59 (5.84)
*Range*	74.83–99.67
**Sex (F; *n*, %)**	56 (72.27%)
**Education (y)**	14.44 (2.66)
**Postmortem interval (h)**	7.09 (3.88)
***APOE* ϵ4 (*n*, %)**	10 (12.99%)
**Diagnosis (*n*, %)**
*CN*	29 (37.66%)
*MCI*	27 (35.07%)
*AD*	21 (27.27%)
**AD-Reagan diagnosis of AD (*n*, %)**
*No to low*	30 (38.96%)
*Intermediate to high*	47 (61.04%)
**LC tangle density**
*Left*	3.48 (3.39)
Right	3.82 (3.73)
**LC neuronal density**
*Left*	77.11 (25.02)
*Right*	75.53 (23.99)

Demographics are provided in mean and standard deviation for continuous variables, or as proportion for categorical variables. AD, Alzheimer’s disease; *APOE*, Apolipoprotein E; LC, locus coeruleus; CN, control; MCI, mild cognitive impairment.

While the neuroimaging literature reported inconsistencies in rostro-caudal asymmetry, we found no evidence for asymmetry in LC pathology between rostral and caudal sections for the entire sample and among individuals with and without AD pathology (Supplementary Table 3), except for a lower neuronal density in the right caudal than left caudal LC section among individuals with AD pathology, relative to the rostral LC (p_Tukey_ = 0.03, Supplementary Figure 3). The TOST (Supplementary Figure 4) showed left-right equivalence across rostro-dorsal sections for LC tangle density up to [– 1,1] and relative tangle density, up to [– 3%, 3% ]. Equivalence bounds of left-right differences in neuronal density across rostro-caudal sections exceeded the smallest meaningful difference and varied between [– 11,11] (entire sample) and [– 19,19] (with AD pathology), indicative of asymmetry as also supported by the repeated measures ANOVA.

## DISCUSSION

Neuropathology studies reported LC neurodegeneration during the course of AD, with modestly increasing asymmetry relative to neurologically healthy individuals [7–9]. Because accumulation of hyperphosphorylated tau in the LC emerges early in adulthood and starts two to three decades prior to neuronal changes [1], the LC has become an important target for early detection of AD, motivating the development of *in vivo* neuroimaging methods of LC integrity [3]. These neuroimaging-based LC metrics covary with tau pathology measured with PET-imaging or blood-based markers [3, 24]. Despite the LC’s early involvement in AD and its critical role in modulating cognition and behavior [3, 5], the clinical relevance of potential asymmetry in LC measures remains unknown. We addressed this gap of knowledge by determining if asymmetry of pathology occurs in the LC across different disease stages, as this will inform the granularity of planned analytical approaches, the interpretation of MRI-based LC findings during AD progression and in the context of clinical heterogeneity. We found that the amount of tangle density as well as relative tangle density were equivalent in the left and right LC, both when considering the rostral or caudal sections separately or when analyzing individuals with or without evidence of underlying AD pathology. However, considering the range of reported percent differences in neuronal count in AD [7], our results indicate that the left versus right neuronal density in the caudal LC is different from the pattern of neuronal density in the rostral LC among individuals with underlying AD pathology.

Interestingly, the caudal LC contains very tightly clustered cells, but relatively fewer large multipolar cells compared to the rostral LC, which consists of a scattered pattern of both large and small cells. During disease progression, the small, fusiform cells in the dorsal-middle LC are most vulnerable to accumulate tau and show neurodegeneration from Braak stage III– IV [1, 7]. Our data indicates that in older individuals in whom the downstream effects of tau may have been unfolding over several decades, particularly in rostral-middle sections, a more symmetrical pattern of rostral neurodegeneration can be observed. We speculate that as the disease progresses, cells in the caudal section degenerate and become more dispersed. Caudal asymmetry in neuronal degeneration (relative to rostral) may thus signal progression to a more advanced disease stage (above Braak stage IV and Thal stage 3) and possibly correspond to late-stage symptoms including motor-related or autonomic dysfunctions – reflective of its projections to the cerebellum and spinal cord affected earlier in Parkinson’s disease [7, 26]. This does not preclude potential asymmetry in neuronal degeneration of the rostral part earlier in life. We were not able to examine this hypothesis, as the age range in this cohort is older than what is typical for observational studies. An older age range is inherent to autopsy studies but can introduce survival biases and limit the generalizability of our findings to younger populations who likely harbor tau pathology in the LC. In addition to examining a broader age span, it would be valuable for future studies to use more comprehensive methods such as unbiased stereological evaluations, relate asymmetries in neuronal density to symptoms and loss of projection density to cortical target regions [27], and to examine if asymmetry in other read-outs of LC function relate to pathologic asymmetry of the LC [28].

Imaging the LC *in vivo* is feasible with dedicated procedures [3, 29, 30], but often the LC seems shorter in length than what is observed in neuropathology studies. This is most likely because the LC’s cylindrical shape widens along the caudal direction resulting in worse caudal signal-to-noise ratio [6]. The findings of this study hold important implications for *in vivo* imaging studies, as they suggest that in individuals with advanced underlying AD pathologic change neurodegenerative measures of the LC should be investigated in detail, preferably considering both sides separately and examining different sections of the LC. In contrast, our null-findings indicate that tau-related measures may not require this level of detail, facilitating clinical translation of these markers.

## Supplementary Material

Supplementary Material

## Data Availability

Data from the MAP are available upon request at www.radc.rush.edu.

## References

[1] Ehrenberg AJ , Nguy AK , Theofilas P , Dunlop S , Suemoto CK , Di Lorenzo Alho AT , Leite RP , Diehl Rodriguez R , Mejia MB , Rub U , Farfel JM , de Lucena Ferretti-Rebustini RE , Nascimento CF , Nitrini R , Pasquallucci CA , Jacob-Filho W , Miller B , Seeley WW , Heinsen H , Grinberg LT (2017) Quantifying the accretion of hyperphosphorylated tau in the locus coeruleus and dorsal raphe nucleus: The pathological building blocks of early Alzheimer’s disease. Neuropathol Appl Neurobiol 43, 393–408. 28117917 10.1111/nan.12387 PMC5642282

[2] Theofilas P , Ehrenberg AJ , Dunlop S , Di Lorenzo Alho AT , Nguy A , Leite REP , Rodriguez RD , Mejia MB , Suemoto CK , Ferretti-Rebustini REL , Polichiso L , Nascimento CF , Seeley WW , Nitrini R , Pasqualucci CA , Jacob Filho W , Rueb U , Neuhaus J , Heinsen H , Grinberg LT (2017) Locus coeruleus volume and cell population changes during Alzheimer’s disease progression: A stereological study in human postmortem brains with potential implication for early-stage biomarker discovery. , Alzheimers Dement 13, 236–246. 27513978 10.1016/j.jalz.2016.06.2362 PMC5298942

[3] Jacobs HIL , Becker JA , Kwong K , Engels-Dominguez E , Prokopiou PC , Papp KV , Properzi M , Hampton OL , D’Oleire Uquillas F , Sanchez JS , Rentz DM , El Fakhri G , Normandin MD , Price JC , Bennett DA , Sperling RA , Johnson KA (2021) In vivo and neuropathology data support locus coeruleus integrity as indicator of Alzheimer’s disease pathology and cognitive decline. Sci Transl Med 13, eabj2511. 34550726 10.1126/scitranslmed.abj2511 PMC8641759

[4] Jacobs HIL , Becker JA , Kwong K , Munera D , Ramirez-Gomez L , Engels-Dominguez N , Sanchez JS , Vila-Castelar C , Baena A , Sperling RA , Johnson KA , Lopera F , Quiroz YT (2023) Waning locus coeruleus integrity precedes cortical tau accrual in preclinical autosomal dominant Alzheimer’s disease. Alzheimers Dement 19, 169–180. 35298083 10.1002/alz.12656 PMC9481982

[5] Ehrenberg AJ , Kelberman MA , Liu KY , Dahl MJ , Weinshenker D , Falgas N , Dutt S , Mather M , Ludwig M , Betts MJ , Winer JR , Teipel S , Weigand AJ , Eschenko O , Hammerer D , Leiman M , Counts SE , Shine JM , Robertson IH , Levey AI , Lancini E , Son G , Schneider C , Egroo MV , Liguori C , Wang Q , Vazey EM , Rodriguez-Porcel F , Haag L , Bondi MW , Vanneste S , Freeze WM , Yi YJ , Maldinov M , Gatchel J , Satpati A , Babiloni C , Kremen WS , Howard R , Jacobs HIL , Grinberg LT (2023) Priorities for research on neuromodulatory subcortical systems in Alzheimer’s disease: Position paper from the NSS PIA of ISTAART. Alzheimers Dement 19, 2182–2196. 36642985 10.1002/alz.12937 PMC10182252

[6] Fernandes P , Regala J , Correia F , Goncalves-Ferreira AJ (2012) The human locus coeruleus 3-D stereotactic anatomy. Surg Radiol Anat 34, 879–885. 22638719 10.1007/s00276-012-0979-y

[7] Chan-Palay V , Asan E (1989) Alterations in catecholamine neurons of the locus coeruleus in senile dementia of the Alzheimer type and in Parkinson’s disease with and without dementia and depression. J Comp Neurol 287, 373–392. 2570794 10.1002/cne.902870308

[8] Chan-Palay V , Asan E (1989) Quantitation of catecholamine neurons in the locus coeruleus in human brains of normal young and older adults and in depression. J Comp Neurol 287, 357–372. 2570793 10.1002/cne.902870307

[9] German DC , Walker BS , Manaye K , Smith WK , Woodward DJ , North AJ (1988) The human locus coeruleus: Computer reconstruction of cellular distribution. J Neurosci 8, 1776–1788. 3367220 10.1523/JNEUROSCI.08-05-01776.1988 PMC6569207

[10] Braak H , Del Tredici K (2015) Neuroanatomy and pathology of sporadic Alzheimer’s disease, Springer International Publishing, Switzerland. 25920101

[11] Engels-Dominguez N , Koops EA , Prokopiou PC , Van Egroo M , Schneider C , Riphagen JM , Singhal T , Jacobs HIL (2023) State-of-the-art imaging of neuromodulatory subcortical systems in aging and Alzheimer’s disease: Challenges and opportunities. Neurosci Biobehav Rev 144, 104998. 36526031 10.1016/j.neubiorev.2022.104998 PMC9805533

[12] Betts MJ , Cardenas-Blanco A , Kanowski M , Jessen F , Duzel E (2017) In vivo MRI assessment of the human locus coeruleus along its rostrocaudal extent in young and older adults. Neuroimage 163, 150–159. 28943414 10.1016/j.neuroimage.2017.09.042

[13] Dahl MJ , Mather M , Duzel S , Bodammer NC , Lindenberger U , Kuhn S , Werkle-Bergner M (2019) Rostral locus coeruleus integrity is associated with better memory performance in older adults. Nat Hum Behav 3, 1203–1214. 31501542 10.1038/s41562-019-0715-2 PMC7203800

[14] Elman JA , Puckett OK , Beck A , Fennema-Notestine C , Cross LK , Dale AM , Eglit GML , Eyler LT , Gillespie NA , Granholm EL , Gustavson DE , Hagler DJ Jr. , Hatton SN , Hauger R , Jak AJ , Logue MW , McEvoy LK , McKenzie RE , Neale MC , Panizzon MS , Reynolds CA , Sanderson-Cimino M , Toomey R , Tu XM , Whitsel N , Williams ME , Xian H , Lyons MJ , Franz CE , Kremen WS (2021) MRI-assessed locus coeruleus integrity is heritable and associated with multiple cognitive domains, mild cognitive impairment, and daytime dysfunction. Alzheimers Dement 17, 1017–1025. 33580733 10.1002/alz.12261 PMC8248066

[15] Cassidy CM , Therriault J , Pascoal TA , Cheung V , Savard M , Tuominen L , Chamoun M , McCall A , Celebi S , Lussier F , Massarweh G , Soucy JP , Weinshenker D , Tardif C , Ismail Z , Gauthier S , Rosa-Neto P (2022) Association of locus coeruleus integrity with Braak stage and neuropsychiatric symptom severity in Alzheimer’s disease. Neuropsychopharmacology 47, 1128–1136. 35177805 10.1038/s41386-022-01293-6 PMC8938499

[16] Jacobs HI , Wiese S , van de Ven V , Gronenschild EH , Verhey FR , Matthews PM (2015) Relevance of parahippocampal-locus coeruleus connectivity to memory in early dementia. Neurobiol Aging 36, 618–626. 25433457 10.1016/j.neurobiolaging.2014.10.041

[17] Mather M , Harley CW (2016) The locus coeruleus: Essential for maintaining cognitive function and the aging brain. Trends Cogn Sci 20, 214–226. 26895736 10.1016/j.tics.2016.01.001 PMC4761411

[18] Bennett DA , Buchman AS , Boyle PA , Barnes LL , Wilson RS , Schneider JA (2018) Religious Orders Study and Rush Memory and Aging Project. J Alzheimers Dis 64, S161–S189. 29865057 10.3233/JAD-179939 PMC6380522

[19] Bennett DA , Schneider JA , Aggarwal NT , Arvanitakis Z , Shah RC , Kelly JF , Fox JH , Cochran EJ , Arends D , Treinkman AD , Wilson RS (2006) Decision rules guiding the clinical diagnosis of Alzheimer’s disease in two community-based cohort studies compared to standard practice in a clinic-based cohort study. Neuroepidemiology 27, 169–176. 17035694 10.1159/000096129

[20] Bennett DA , Wilson RS , Schneider JA , Evans DA , Beckett LA , Aggarwal NT , Barnes LL , Fox JH , Bach J (2002) Natural history of mild cognitive impairment in older persons. Neurology 59, 198–205. 12136057 10.1212/wnl.59.2.198

[21] Bennett DA , Schneider JA , Arvanitakis Z , Kelly JF , Aggarwal NT , Shah RC , Wilson RS (2006) Neuropathology of older persons without cognitive impairment from two community-based studies. Neurology 66, 1837–1844. 16801647 10.1212/01.wnl.0000219668.47116.e6

[22] Buchman AS , Nag S , Shulman JM , Lim AS , VanderHorst VG , Leurgans SE , Schneider JA , Bennett DA (2012) Locus coeruleus neuron density and parkinsonism in older adults without Parkinson’s disease. Mov Disord 27, 1625–1631. 23038629 10.1002/mds.25142 PMC3628555

[23] Wilson RS , Nag S , Boyle PA , Hizel LP , Yu L , Buchman AS , Schneider JA , Bennett DA (2013) Neural reserve, neuronal density in the locus ceruleus, and cognitive decline. Neurology 80, 1202–1208. 23486878 10.1212/WNL.0b013e3182897103 PMC3691778

[24] Van Egroo M , Riphagen JM , Ashton NJ , Janelidze S , Sperling RA , Johnson KA , Yang HS , Bennett DA , Blennow K , Hansson O , Zetterberg H , Jacobs HIL (2023) Ultra-high field imaging, plasma markers and autopsy data uncover a specific rostral locus coeruleus vulnerability to hyperphosphorylated tau. Mol Psychiatry 28, 2412–2422. 37020050 10.1038/s41380-023-02041-y PMC10073793

[25] Lakens D , Scheel AM , Isager PM (2018) Equivalence testing for psychological research: A tutorial. Adv Methods Pract Psychol Sci 1, 259–269.

[26] O’Callaghan C , Hezemans FH , Ye R , Rua C , Jones PS , Murley AG , Holland N , Regenthal R , Tsvetanov KA , Wolpe N , Barker RA , Williams-Gray CH , Robbins TW , Passamonti L , Rowe JB (2021) Locus coeruleus integrity and the effect of atomoxetine on response inhibition in Parkinson’s disease. Brain 144, 2513–2526. 33783470 10.1093/brain/awab142 PMC7611672

[27] Gilvesy A , Husen E , Magloczky Z , Mihaly O , Hortobagyi T , Kanatani S , Heinsen H , Renier N , Hokfelt T , Mulder J , Uhlen M , Kovacs GG , Adori C (2022) Spatiotemporal characterization of cellular tau pathology in the human locus coeruleus-pericoerulear complex by three-dimensional imaging. Acta Neuropathol 144, 651–676. 36040521 10.1007/s00401-022-02477-6 PMC9468059

[28] Liu Y , Rodenkirch C , Moskowitz N , Schriver B , Wang Q (2017) Dynamic lateralization of pupil dilation evoked by locus coeruleus activation results from sympathetic, not parasympathetic, contributions. Cell Rep 20, 3099–3112. 28954227 10.1016/j.celrep.2017.08.094 PMC5679481

[29] Priovoulos N , Jacobs HIL , Ivanov D , Uludag K , Verhey FRJ , Poser BA (2018) High-resolution *in vivo* imaging of human locus coeruleus by magnetization transfer MRI at 3T and 7T. Neuroimage 168, 427–436. 28743460 10.1016/j.neuroimage.2017.07.045

[30] Van Egroo M , van Hooren RWE , Jacobs HIL (2021) Associations between locus coeruleus integrity and nocturnal awakenings in the context of Alzheimer’s disease plasma biomarkers: A 7T MRI study. Alzheimers Res Ther 13, 159. 34560904 10.1186/s13195-021-00902-8 PMC8464124

