# Supplementary material for: Sparse Asymmetry in Locus Coeruleus Pathology in Alzheimer’s Disease
Source: J Alzheimers Dis. 2024 Apr 30;99(1):105–11. doi: 10.3233/JAD-231328 (PMC11091606; doi:10.3233/JAD-231328)
Supplement: Supplementary Material [file jad-99-jad231328-s001.pdf]

Supplementary Material

Sparse Asymmetry in Locus Coeruleus Pathology in Alzheimer's Disease

Supplementary Table 1. Association between left-right differences in locus coeruleus pathology measures and age, sex or postmortem interval

Hemisphere x covariate	F-value	p	Effect size (partial η^2)
**Interactions with age as between subject factor (n=77)**			
Tangle density asymmetry by age	0.015	0.90	<0.001
Neuronal density asymmetry by age	0.381	0.54	0.005
Relative tangle density asymmetry by age	0.109	0.74	0.001
**Interactions with sex as between subject factor (n=77)**			
Tangle density asymmetry by sex	0.001	0.99	<0.001
Neuronal density asymmetry by sex	0.294	0.59	0.003
Relative tangle density asymmetry by sex	0.163	0.69	0.002
**Interactions with postmortem interval (pmi) as between subject factor (n=77)**			
Tangle density asymmetry by pmi	0.506	0.95	0.75
Neuronal density asymmetry by pmi	0.808	0.72	0.83
Relative tangle density asymmetry by pmi	0.725	0.80	0.81

Repeated measures ANOVA with hemisphere (left, right) as repeated levels (with Greenhous-Geiser correction) in interaction with age or sex or postmortem interval time (between-subject level).

Supplementary Figure 1. Flow chart of the statistical analyses

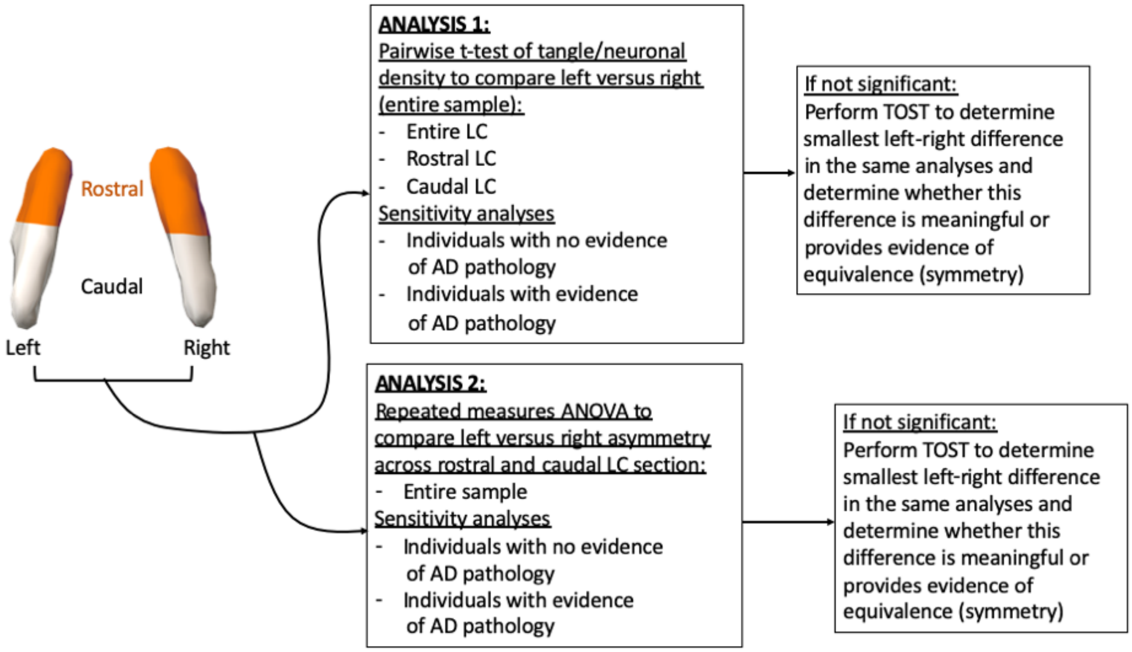

Schema depicting the key analyses in the manuscript. Pairwise t-test were adjusted for multiple comparisons using the False Discovery Rate. Repeated measures ANOVA were adjusted using Tukey.

Supplementary Table 2. Left-right differences in locus coeruleus pathology measures

	Mean difference	T-value (df=76)	p
**Entire sample (N=77)**			
**Entire LC**			
Tangle density	-0.34	-1.61	0.11
Neuronal density	1.58	0.89	0.38
Relative tangle density	-0.56	-1.80	0.075
**Rostral LC**			
Tangle density	-0.61	-1.84	0.07
Neuronal density	-1.39	-0.56	0.57
Relative tangle density	-1.25	-1.66	0.10
**Caudal LC**			
Tangle density	-0.06	-0.23	0.82
Neuronal density	4.55	1.75	0.08
Relative tangle density	-0.11	-0.25	0.80
**Sensitivity analyses: Without evidence of AD pathology (N=30)**			
**Entire LC**			
Tangle density	0.27	0.83	0.42
Neuronal density	0.52	0.19	0.85
Relative tangle density	-0.18	-0.37	0.71
**Rostral LC**			
Tangle density	-0.5	-1.07	0.29
Neuronal density	0.8	0.22	0.83
Relative tangle density	-0.54	-0.55	0.59
**Caudal LC**			
Tangle density	-0.03	-0.08	0.94
Neuronal density	0.23	0.06	0.96
Relative tangle density	0.57	0.82	0.42
**Sensitivity analyses: With evidence of AD pathology (N=47)**			
**Entire LC**			
Tangle density	-0.38	-1.37	0.18
Neuronal density	2.27	0.97	0.34
Relative tangle density	-0.80	-1.98	**0.05**
**Rostral LC**			
Tangle density	-0.68	-1.48	0.15
Neuronal density	-2.79	-0.85	0.40
Relative tangle density	-1.70	-1.59	0.12
**Caudal LC**			
Tangle density	-0.09	-0.22	0.82
Neuronal density	7.32	2.24	**0.03**
Relative tangle density	-0.54	-1.07	0.29

Observed left-right differences for the entire LC, rostral LC and caudal LC and for the tangle density, neuronal density and relative tangle density measures within the entire sample, individuals with and without evidence of underlying AD pathology according the NIA-Reagan criteria for AD diagnosis. Paired one sample t-tests were performed. Tangle and neuronal density expressed per mm^2 and relative tangle density as a percentage.

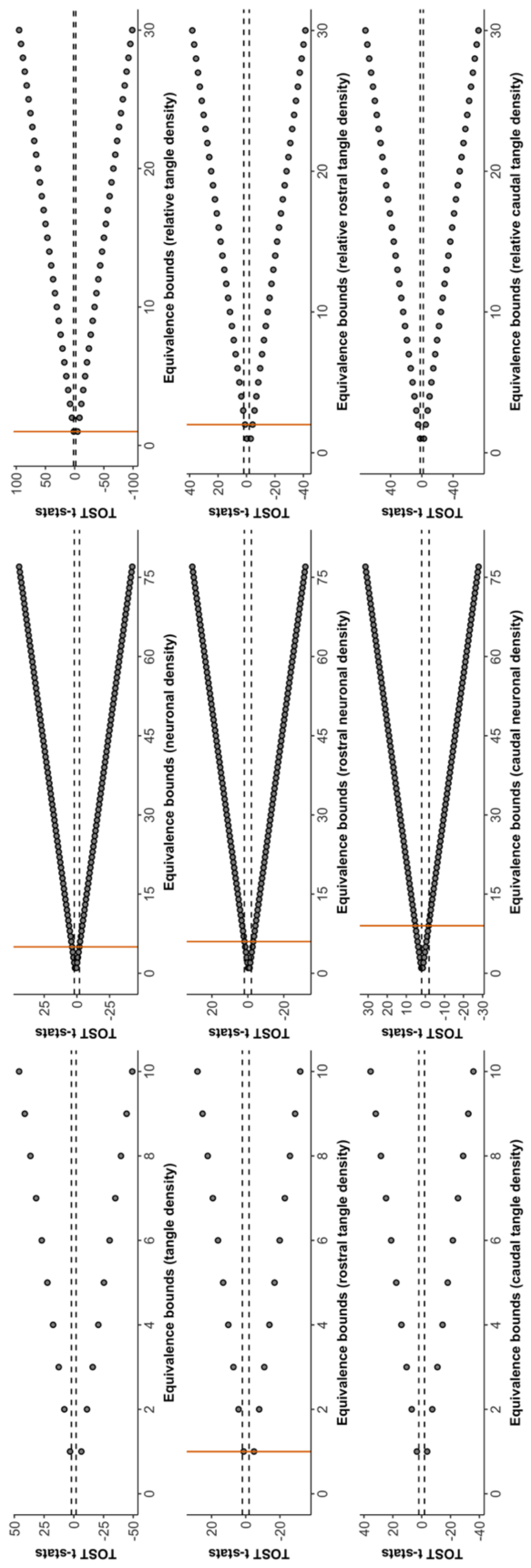

Supplementary Figure 2. Iterative evaluation of left-right equivalence bounds of LC pathology

Graphs depict the TOST t-statistics (upper and lower) at different equivalence bounds for each LC measure. Equivalence bounds were gradually decreased from the maximum observed difference between left and right to detect the highest bound (red line) where equivalence was no longer significant. Significance threshold are indicated with the dashed lines at $\alpha=0.05$. Analyses were run for the entire LC (top row), rostral LC (middle row) and caudal LC (bottom row) and for the tangle density (left column), neuronal density (middle column) and relative tangle density measures (right column).

Supplementary Table 3. Left-right differences between the rostral and caudal sections of the LC

Hemisphere x section	F-value	p	Effect size (partial η^2)
**Entire sample (n=77)**			
Tangle density	1.48	0.23	0.019
Neuronal density	2.76	0.10	0.035
Relative tangle density	1.75	0.19	0.023
**Sensitivity analyses: Without evidence of AD pathology (n=30)**			
Tangle density	0.59	0.45	0.020
Neuronal density	0.01	0.92	0.000
Relative tangle density	0.95	0.34	0.032
**Sensitivity analyses: With evidence of AD pathology (n=47)**			
Tangle density	0.89	0.35	0.019
Neuronal density	4.83	**0.03**	0.095
Relative tangle density	0.90	0.35	0.019

Repeated measures ANOVA with hemisphere (left, right) and section (rostral, caudal) as repeated levels (with Greenhous-Geiser correction) within the entire sample, individuals with and without evidence of underlying AD pathology according the NIA-Reagan criteria for AD diagnosis. Results are adjusted for multiple corrections using Tukey.

Supplementary Figure 3. Larger left-right asymmetry in caudal than rostral LC neuronal density in individuals with underlying AD pathology (n=47)

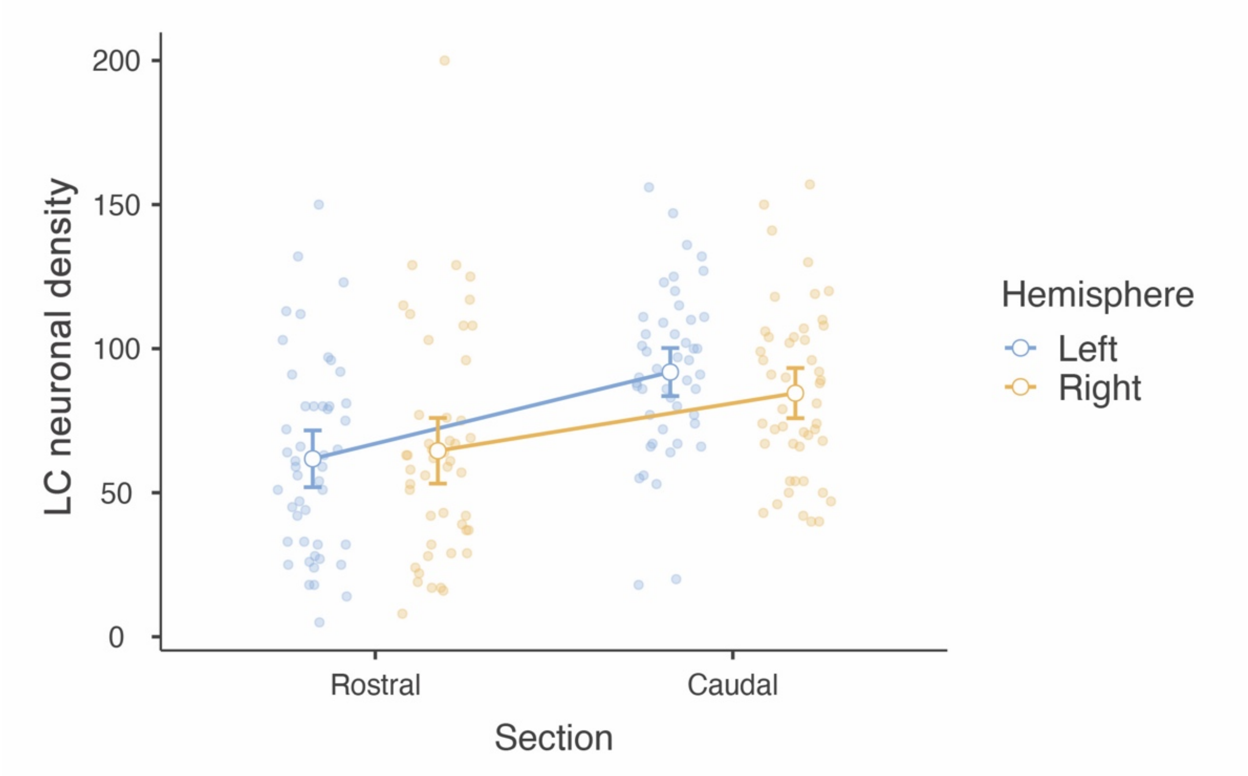

Among individuals with underlying evidence of AD pathology, a larger left-right asymmetry of neuronal density in the caudal section was observed relative to left-right asymmetry in the rostral section of the LC (medium effect size of partial η^2=0.095; Neuronal density mean of rostral LC: left: 61.8, right: 64.6; caudal LC: left: 91.9 and right: 84.6).

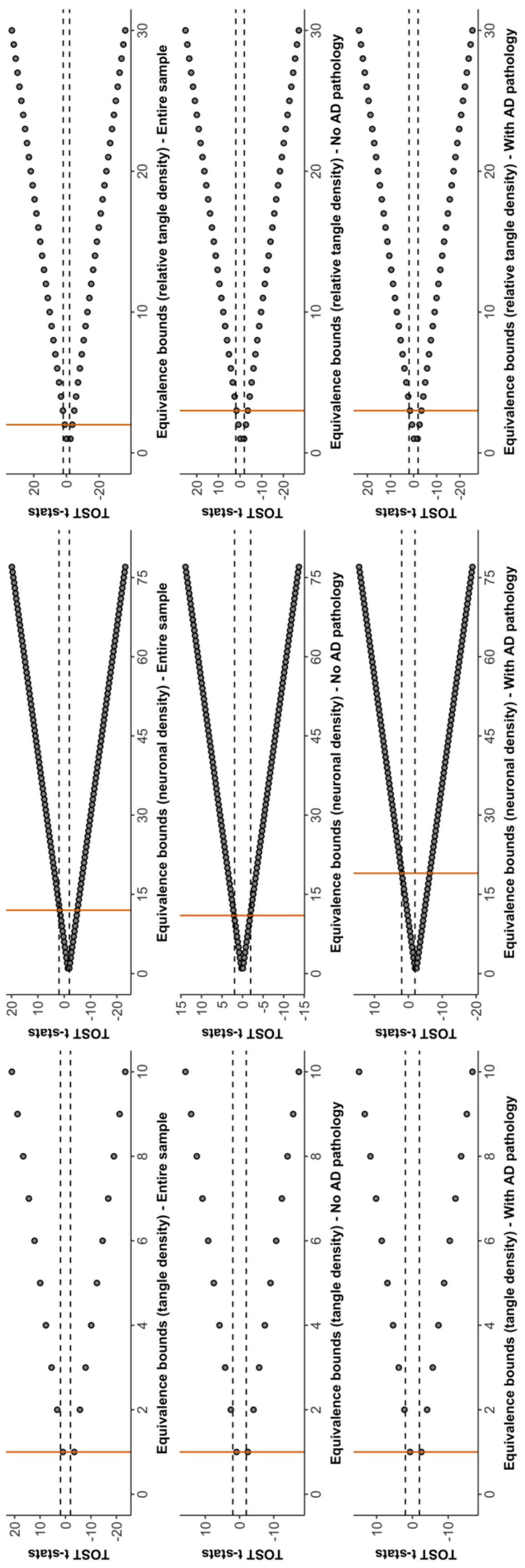

Supplementary Figure 4. Iterative evaluation of equivalence bands for left-right asymmetry between rostral and caudal LC sections

Graphs depict the TOST t-statistics (upper and lower) at different equivalence bounds for each LC measure (testing whether left-right asymmetry in the rostral portion of the LC is equivalent to that in the caudal portion of the LC). Equivalence bounds were gradually decreased from the maximum observed difference between left and right to detect the highest bound (red line) where equivalence was no longer significant. Significance threshold are indicated with the dashed lines at α=0.05. Analyses were run for the entire LC, rostral LC and caudal LC and for the tangle density, neuronal density and relative tangle density measures. Top row depicts the entire group, middle row the group without AD pathology (sensitivity analyses) and the bottom row the group with AD pathology (sensitivity analyses).
